# Prevalence of Sexual Dysfunctions Among Women with Multiple Sclerosis

**DOI:** 10.1007/s11195-013-9293-9

**Published:** 2013-03-28

**Authors:** M. Lew-Starowicz, R. Rola

**Affiliations:** 1 III Department of Psychiatry, Institute of Psychiatry and Neurology, Warsaw, Poland; 2 I Department of Neurology, Institute of Psychiatry and Neurology, Warsaw, Poland; 3 Department of Human Physiology and Pathophysiology, The Medical University of Warsaw, Warsaw, Poland

**Keywords:** Multiple sclerosis, Female sexual dysfunction, Prevalence, SFQ28, Poland

## Abstract

Sexual concerns are known to be common in women suffering from multiple sclerosis (MS) but definite data on the prevalence of particular sexual dysfunctions (SD) remain unclear. Previous studies brought inconsistent findings and rely on small groups of patients or use of unvalidated assessment methods. The aim of this research was to evaluate the prevalence of SD in women with MS using validated clinimetric scales. 137 female inpatients with MS diagnosis were interviewed, completed The Female Sexual Function Questionnaire SFQ28 and underwent neurological assessment. Only 2.2 % of patients had ever discussed their sexual concerns with a physician. 70.1 % reported sexual activity. At least one SD could be found in 82.5 % of patients, hypoactive sexual desire (57.7 %), arousal dysfunction (decreased genital sensation in 47.3 %, decreased lubrication in 48.4 %, decreased subjective arousal in 45.2 %) and orgasmic dysfunction (39.8 %) being the most probable. SD were less likely in women who assessed their relationship positively but more common in older patients and those who had a positive history of depression. The prevalence of SD was higher comparing to the majority of studies by other authors. In conclusion, SD are very common in female patients with MS and permanently overlooked by medical professionals. Therefore, the assessment of sexual function should be implemented in all patients after the diagnosis of MS. Further research is needed for better understanding of the sexuality of this particular population in order to establish targets for therapeutic intervention.

## Introduction

Sexual activity has had a strong impact when it comes to determining a basic quality of life, self-esteem, self-image and quality of interpersonal relationships. According to research in this area, individuals with sexual dysfunctions (SD) have a diminished quality of life compared to others and show poorer outcomes in all major dimensions determining wellbeing like health, achievements, personal relationships, safety and a feeling of being a part of their community [1]. Based on research by McCabe and Taleporos on 1,196 subjects, it was demonstrated that people who had more severe physical impairments and who had significantly lower levels of sexual esteem and sexual satisfaction were sexually active less frequently and more depressed than able-bodied people [2]. The negative influence of a set of comorbidities such as depression, cardiovascular disease and diabetes mellitus on sexual function is well known [3–7]. Sexual concerns of patients suffering from a neurological disease (especially polyneuropathies and myelopathies) are common [8, 9], but still remain poorly understood in clinical practice.

It has been known for a long time that SD in women with multiple sclerosis (MS) are more prevalent than in the general population [10]. They are thought to be derived from disease-related neurologic changes—demyelination and the atrophy of nerve fibers that impact sexual response (primarily SD), fatigue and physical disability related to MS (secondary SD). This would also apply to psychological and sociocultural aspects of a chronic disease (tertiary SD) [11, 12]. The disease-specific neurologic lesions affecting sexual activity can be localized in the brain, spinal cord or peripheral neurons. Autonomic fiber injuries can also cause SD accompanied by bladder and bowel incontinence that contribute to physical discomfort (i.e. fear from unwanted urination or defecation) during intercourse [10, 13]. In a recent study of 19 men and 52 women with MS by Khan and colleagues [14] significant negative correlations between the Total Personal Experiences Questionnaire (PEQ) Sexual Frequency scale and Neurological Disability Scale (ρ = −0.30) were found. The scores in the PEQ Sexual Frequency scale also correlated negatively with the Quality of life item from the American Urological Association Bladder score (ρ = −0.35) and with the MS Impact Scale Physical subscale (ρ = −0.30). Khan and colleagues concluded that the demyelinating lesions causing physical impairments and bladder dysfunction in MS may also affect SD.

Symptoms of weakness of the pelvic floor, bladder and bowel dysfunction are correlated with changes in lubrication and orgasmic capacity. Lesions in the sacral segments of the spinal cord that affect the sacral reflex arc, clitoral and vaginal sensory deficits may cause decreased physical arousal (lubrication, sensations) and anorgasmia. Spasticity, impaired mobility and coordination, fatigue, pain, numbness and weakness of pelvic floor muscles are other factors that affect satisfactory intercourse [15–17]. The tertiary SD (psychological and sociocultural aspects) include negative changes in mood (depressive mood) and self-image, feeling less attractive, changing gender roles and roles in the couple, difficulties in communicating with one’s partner, feelings of guilt, dependency and fear of being sexually rejected, abandoned or isolated [11]. Nearly half of all patients with MS report mental comorbidity along with depression as the most common—an estimated 46 % in a study of 8,983 MS patients by Marrie and colleagues [18].

SD in women with MS as well as other neurological diseases was further investigated by several authors. However, most studies rely on small groups of patients and author-designed interviews or questionnaires. The frequency of SD in women with MS was recently reviewed by Bronner and colleagues [19] and a wide variety of studies by different authors indicate significant differences in estimation of decrease in sexual desire (31.4–63.6 %) [13, 20–24] and impaired arousal (33.0–51.5 %) [20, 22–25]. However, this was seen to a much lesser extent when it came to evaluating orgasmic difficulties (37.0–38.3 %) [20, 22, 23].

Using unvalidated questionnaires in SD epidemiological studies is especially problematic as the definition and criteria of SD often remain unclear. Classifications introduced by the World Health Organization (ICD-10) [26] and the American Psychiatric Association (DSM-IV) [27] have created definitions for SD based mainly upon the physiological model of genital responses and symptoms that serve to hinder coital intercourse. Both sets regard SD involving various combinations of physical and psychological constituents. However, it remains controversial that they believe it is possible to separate these [28]. For epidemiological purposes, it is important to use a well-recognized and validated set of screening tools that involve both physical and psychological aspects of SD. This will allow for the creation of comparable databases of SD in certain populations that could be further analyzed in order to draw clinical implications.

The aim of our research was to accurately evaluate the prevalence of SD in women with MS. To our knowledge, our group is the largest sample of women with MS investigated for SD in a single study with a validated questionnaire.

## Material and Methodology

One hundred and thirty-seven women with MS have been included in the study. The diagnosis was established according to the McDonald`s diagnostic criteria for MS. These criteria require evidence for at least two episodes of neurological dysfunction (dissemination in time, DIT) with the involvement of two different brain regions (dissemination in space, DIS) and MRI confirmation of substantial numbers of demyelination foci in the central nervous system [29]. All of the patients fulfilled the diagnostic criteria for the MS and were patients of the National Multiple Sclerosis Center in Dabek, Poland. Mean disease duration time was 16.4 with a range of 3–38 years. The initial criterion for inclusion involved a definite diagnosis of MS. After this, patients were informed about the study methodology as well as its hypotheses and goals. Patients were then invited to ask questions about the study where all of their concerns were clarified. Only adult patients (age ≥ 18) who had written informed consent were included. Patients who did not want to participate in the study or could not give written informed consent were excluded.

Demographic data were obtained from semi-structured interviews and medical chart review. The information that was collected included age, the onset of symptoms of MS, comorbidities, concomitant medications, a history of depression (clinical diagnosis) and its treatment, marital status and basic obstetric history. Patients were asked whether their sexual life worsened since the onset of MS. They also assessed their current relationship by choosing a description that best fits their perception: definitely negative, rather negative, neutral, somewhat positive or definitely positive. Sexual activity was defined as an activity that may lead to sexual arousal or sexual enjoyment that occurred during a 1 month period before admission to the Rehabilitation Center. It included sexual intercourse, caressing and masturbation.

Patients also completed The Female Sexual Function Questionnaire (SFQ28). The results of it were further discussed with patients in order to ensure that the information obtained was complete and reliable. SFQ28 is a self-report outcomes measure and addresses all aspects of the female sexual response cycle (desire, arousal, orgasm) and pain during sexual activity, according to DSM-IV diagnostic criteria. It also contains questions concerning their sexual enjoyment and relationship with a partner. It has also been used in research regarding female sexual dysfunction (FSD) [30, 31]. The psychometric properties of the SFQ28 were recently assessed by Symonds and colleagues [32] in female sexual arousal disorder (FSAD) candidates and in hypoactive sexual desire disorder (HSDD) populations. The factor analysis confirmed the factor structure of the questionnaire. The SFQ28 demonstrated excellent internal consistency with the Cronbach’s alpha coefficient for each of the domains ranging from 0.70 to 0.93 for subjects with FSAD and from 0.72 to 0.90 for subjects with HSDD. The test–retest reliability was also very strong for each domain involving subjects with FSAD and HSDD. SFQ28 also had good convergent validity with other scales like the Female Sexual Distress Scale (FSDS) and the Sexual Quality of Life-Female (SQOL-F) scale for all domains except pain. The SFQ is recommended as the optimal screening tool for female sexual dysfunction [33] and the Polish version of the questionnaire was recently validated with an estimated reliability of level 0.97 (Cronbach’s alpha test) [34]. In our study, the alpha coefficient value was 0.98.

The SFQ28 contains 28 items and each item represents five or seven possible response options. Results of the SFQ28 are presented in seven domains: Desire, Arousal (sensation), Arousal (lubrication), Arousal (cognitive), Orgasm, Pain, Enjoyment and Partner. Desire domain (six items), addresses pleasurable thoughts and feelings concerning sexual activity, a need to be intimately touched, to participate in sexual activity, initiate sexual activity, and sexual frequency and willingness for sexual activity. Arousal—sensation (four items) refers to feelings of warmth and pulsation within the genital area, and their intensity. Arousal—lubrication (two items) is considered when analyzing the frequency and intensity of vaginal wetness during sexual activity. Arousal—cognitive domain (two items) also reflect the frequency and intensity of emotional sexual arousal (feeling excited, “turned on” etc.). Orgasm domain (three items) concerns the frequency, ease and pleasure of having an orgasm during sexual activity. Pain domain (three items) concerns the frequency and intensity of experiencing pain in the vagina or genital area during or after sexual activity, as well as any anxiety related to pain. The enjoyment domain (six items) reflects enjoyable feelings of being sexually touched or caressed by the partner, enjoyment of sexual activity with or without penetration, feelings of emotional closeness to the partner when taking part in sexual activity, and feeling good about herself and confident as a partner during sexual activity. Partner domain (two items) refers to a patient’s worries regarding how negatively they perceive their partner feels during sexual activity and their level of insecurity in keeping their relationship due to problems in their sexual life [31, 34]. The higher score indicates a better sexual function. Scores for the categories of: Desire, Arousal, Orgasm, Pain and Enjoyment are further subdivided into three categories that include: a high probability of FSD, borderline sexual function and high probability of normal sexual function (Table 1) [30, 31]. There are also no score ranges for Partner domain and for a total score of the SFQ28. Higher scores are interpreted as a better assessment of the relationship and indicate less sexual dysfunction, respectively.

**Table 1:** SFQ28 scores indicating probability of sexual dysfunction

Domain	Scores indicating high probability of FSD	Scores indicating borderline sexual function	Scores indicating high probability of normal sexual function
Desire	5–16	17–22	23–31
Arousal (S)	4–10	11–13	14–20
Arousal (L)	2–5	6–7	8–10
Arousal (C)	2–5	6–7	8–10
Orgasm	1–8	9–11	12–15
Pain	2–8	9–11	12–15
Enjoyment	6–16	17–22	23–30

Arousal Domains, *S* sensation, *L* lubrication, *C* cognitive

An Expanded Disability Status Scale (EDSS) was performed by the one and only independent rater (neurologist) according to the method described originally by John Kurtzke [35]. EDSS is the most frequently used scale when it comes to the assessment and quantification of symptoms’ severity and physical functioning of patients with MS. EDSS scoring is based on measuring the everyday activities (ambulation with or without walking aids, walking distance, need for assistance and help with walking), physical functioning and neurological symptoms, such as: pyramidal, cerebellar, brainstem, sensory, bowel and bladder, visual and psychiatric dysfunction. A final score is presented numerically on a scale of 0–10 where 0 stands for normal functioning without any neurological deficits and 10 represents “death due to MS”. Therefore, EDSS covers both the physical functioning and severity of the symptoms for patients with MS [35]. Three (3) out of 137 patients refused to participate in the neurological evaluation. Furthermore, an independent rater did not have any insight into their medical history, observation, type of the disease course, disease duration, and results of their sexual function evaluation.

Statistical analysis was performed using a Graph Pad In Stat 3.06 for Windows and SPSS 20.0. Frequencies were then calculated for each variable. The data was presented in the paper using means and standard deviation. Values sampled from normal (Gaussian) distributions were appropriately compared using a Student’s *t* test and ANOVA measures followed by Tukey post hoc tests. Values without normal distributions were compared by nonparametric tests: Mann–Whitney-*U* test and Kruskal–Wallis test followed by a Dunn post hoc test. Only measures with a *p* < 0.05 were considered statistically significant.

## Results

One hundred and thirty-seven women with MS completed the study. The mean age of participants was 50.7 ± 7.0, disease duration 16.4 ± 8.6, age of MS onset 34.7 ± 9.2 and relationship duration 24.5 ± 10.5 years. The mean EDSS score was 5.2 ± 0.2 points. 94 patients reported concomitant morbidities where the most frequent were: history of depression (30 patients), hypertension (15) and osteoarthritis (10).

**Fig. 1:**
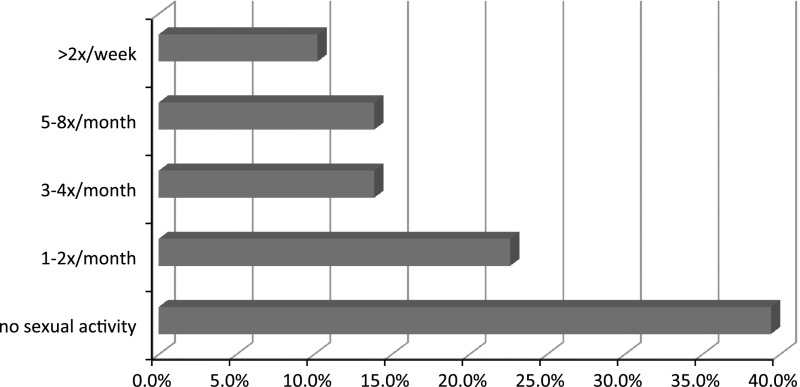
Sexual activity of women with MS during last month before examination

Sexual activity was reported by 96 women (70.1 %). Among patients living in stable relationships, 85 % were sexually active compared to (0 %) of single and widowed women. Typical frequency of sexual activity is shown in Fig. 1. Only nine (6.6 %) of the patients reported masturbating. All of them were in stable relationships, and seven (77.8 %) had intercourse with their partners. Most of the women perceived their relationship as a positive experience: (40.2 % as definitely positive and 41.1 % as somewhat positive, respectively), 10.7 % were neutral, 5.4 % were rather negative and only one person (0.9 %) reported a definitely negative experience. Lastly, 1.8 % of the patients did not respond to the questions.

The majority of all of the subjects (n = 121, 88.3 %) had children and 18 (13.1 %) had a history of miscarriage (83.3 % occurred after the onset of MS). Only 43 (31.4 %) were pregnant and 32 (23.4 %) gave birth after the onset of MS.

The data from SFQ28 are presented in Table 2. The percentages of high probability of FSD, borderline SF and the high probability of normal SF in Partner Domain and total score of SFQ28 are marked not available as there are no reference score ranges for these domains. At least one sexual dysfunction (defined as high probability of FSD according to scores in Desire, Arousal, Orgasm or Pain Domain of the SFQ28) could be found in 113 (82.5 %) of patients. About a half of the women (50.6 %) with a high probability of HSDD according to SFQ28 were sexually active with their partners. The comparison performed with Mann–Whitney-*U* test has shown that sexually active patients scored, on average, much higher in the Desire Domain than those who denied having intercourse (16.9 ± 6.1 vs 8.6 ± 4.2, respectively) indicating a significantly better sexual function (*p* < 0.0001).

**Table 2:** Sexual function in women with MS measured in SFQ28

Measure	SFQ28 domain								
	Desire	Arousal (sensation)	Arousal (lubrication)	Arousal (cognitive)	Orgasm	Pain	Enjoyment	Partner	Total score
Mean	14.4	11.2	5.5	5.6	7.9	13.3	17.8	8.7	91.8
Standard deviation	6.8	4.1	2.1	2.0	4.3	2.5	5.9	1.8	20.1
Standard error	0.6	0.4	0.2	0.2	0.4	0.3	0.6	0.2	2.2
Median	15.0	11.0	6.0	6.0	9.0	15.0	19.0	10.0	93.0
n	137	91	93	93	93	88	95	135	81
HP FSD n (%)	79 (57.7 %)	43 (47.3 %)	45 (48.4 %)	42 (45.2 %)	37 (39.8 %)	5 (5.7 %)	34 (35.8 %)	N/A	N/A
Borderline SF n (%)	40 (29.2 %)	25 (27.5 %)	32 (34.4 %)	34 (36.6 %)	38 (40.9 %)	12 (13.6 %)	40 (42.1 %)	N/A	N/A
HP NSF n (%)	18 (13.1 %)	23 (25.3 %)	16 (17.2 %)	17 (18.3 %)	18 (19.4 %)	71 (80.7 %)	21 (22.1 %)	N/A	N/A

*n* sample size, *HP FSD* high probability of female sexual dysfunction, *borderline SF* borderline sexual function, *HP NSF* high probability of normal sexual function, *N/A* not available

Less than half of the subjects (41.5 %) claimed that their sexual life worsened. For 55.4 % it did not change, and for only 3.1 % it improved after the onset of MS. Women who reported no change in their sexual life since the onset of MS scored significantly better in SFQ28 than those who perceived that it had worsened: 16.3 ± 6.9 versus 12.4 ± 5.5 in Desire (*p* < 0.005; Mann–Whitney-*U* test), 11.8 ± 3.6 versus 9.9 ± 4.5 in Arousal—sensation (*p* < 0.05; Student-*t* test), 6.0 ± 2.0 versus 4.5 ± 2.0 in Arousal—lubrication (*p* < 0.005; Mann–Whitney-*U* test), 8.7 ± 3.9 versus 6.25 ± 4.3 in Orgasm (*p* < 0.005; Mann–Whitney-*U* test), 18.9 ± 5.3 versus 15.4 ± 5.9 in Enjoyment (*p* = 0,005; Mann–Whitney-*U* test) and 95.0 ± 18.8 versus 83.1 ± 19.8 in SFQ28 total score (*p* < 0.01; Mann–Whitney-*U* test). The differences were not quite as significant in Arousal—cognitive (5.8 ± 1.8 vs. 5.0 ± 2.2, respectively, *p* = 0.084; Mann–Whitney-*U* test) and Partner Domain (9.0 ± 1.7 vs. 8.3 ± 2.0, respectively, *p* = 0,055; Mann–Whitney-*U* test). The difference in the Pain Domain was insignificant.

A comparison of sexual function between age groups was shown in Table 3. The most significant were the differences between the youngest (≤ 45 y.o.) and the oldest (≥ 56 y.o.) group of patients in the categories of Desire and Arousal, as well as in their SFQ28 total score in favor of the youngest. Patients with a positive history of depression (23.4 %) scored significantly lower than the others in Desire (11.4 ± 6.2 vs. 15.3 ± 6.7, *p* < 0.005; Mann–Whitney-*U* test), Arousal—sensation (9.5 ± 4.9 vs. 11.6 ± 3.8, *p* < 0.05; Student-*t* test) and Enjoyment (14.7 ± 7.6 vs. 18.5 ± 5.2, *p* < 0.01; Mann–Whitney-*U* test) domains of SFQ28.

**Table 3:** Comparison of SFQ28 scores between age groups in women with MS

SFQ28	≤ 45	46–55	≥ 56
Domain score	Age group	Age group	Age group
Desire**	16.6 ± 6.6	15.0 ± 6.6	11.7 ± 6.4
Arousal (sensation)*	12.5 ± 3.6	11.5 ± 3.7	9.3 ± 4.6
Arousal (lubrication)**	6.6 ± 2.1	5.5 ± 1.9	4.5 ± 2.2
Arousal (cognitive)**	6.5 ± 1.8	5.7 ± 1.8	4.5 ± 2.2
Orgasm*	8.7 ± 4.4	8.3 ± 4.0	6.2 ± 4.4
Pain*	14.3 ± 1.5	13.3 ± 1.9	12.3 ± 3.6
Enjoyment	19.3 ± 5.3	18.1 ± 5.8	15.3 ± 6.2
Partner	8.5 ± 1.9	9.0 ± 1.6	8.5 ± 2.0
SFQ28 total score*	99.4 ± 17.8	92.4 ± 17.7	81.0 ± 23.9

The scores are presented as means ± standard deviations

* Statistical significance *p* < 0.05 between groups

** Statistical significance *p* < 0.005 between groups

The analyses were performed using Kruskal–Wallis nonparametric test followed by Dunn post hoc test except the comparison in Arousal-sensation domain which was performed using ANOVA followed by Tukey post hoc test

The assessment of relationship experience in women with MS did not vary according to their age or EDSS score (*p* > 0.05), whereas SFQ28 scores were clearly different in patients who assessed their relationships differently (Table 4). Only 3 (2.2 %) of all patients have ever tried sexual or couple therapy including discussing their sexual issues with a physician, psychologist, or physiotherapist.

**Table 4:** SFQ28 scores according to relationship assessment

	Relationship assessment		
	Negative/neutral	Somehow positive	Very positive
Desire**	12.9 ± 5.6	14.4 ± 6.5	18.3 ± 5.8
Arousal (sensation)**	8.9 ± 4.3	10.0 ± 3.4	13.0 ± 3.9
Arousal (lubrication)*	4.2 ± 1.7	5.5 ± 2.2	6.0 ± 2.1
Arousal (cognitive)*	4.4 ± 2.25	5.3 ± 1.7	6.3 ± 2.0
Orgasm**	4.1 ± 3.4	7.8 ± 3.8	9.4 ± 4.1
Enjoyment**	11.7 ± 4.8	17.9 ± 5.2	20.5 ± 4.6
Pain*	12.2 ± 3.3	13.0 ± 2.5	14.0 ± 1.9
Partner	8.3 ± 2.3	8.7 ± 1.7	9.2 ± 2.3
SFQ28 total score**	68.8 ± 18.3	90.8 ± 15.8	98.3 ± 19.4

The scores are presented as means ± standard deviations

* Statistical significance *p* < 0.05 between groups

** Statistical significance *p* < 0.005 between groups

SFQ28 scores are presented as means ± standard deviations. The scores in Arousal (sensation) and Enjoyment domains, and SFQ28 total score were compared within groups using ANOVA followed by Tukey post hoc tests. Other domains were compared using Kruskal–Wallis nonparametric test followed by Dunn post hoc test

## Discussion

In the literature there are many studies on different health related subjects connected to MS but very few facing sexual issues [36]. To date, research that uses large cohorts and validated tools is specific to men with MS and relates mostly to erectile dysfunction. This is surprising, as MS affects women more often than men. A possible explanation for this is the influence of the pharmaceutical industry and the high demand for phosphodiesterase-5 inhibitors that catalysed studies on male SD and a search for target populations. Moreover, according to Nortvedt and colleagues [37], men with MS can be much more dissatisfied with their sexual functions than women with MS. This also makes them an easier target for research. On the other hand, the studies on FSD were conducted for smaller groups often using a less than reliable methodology (typically author-designed, short questionnaires). Our study was intended to fill the gap in the research on sexual functioning in women with MS. Another advantage is our patient sample size and use of reliable methodology for the assessment of sexual function.

The vast majority (97.8 %) of our patients had neither tried sexual or couple therapy nor discussed their sexual concerns with any physician, psychologist or physiotherapist. This could be related to a relatively weak knowledge about sexual dysfunctions (SD) among medical care professionals and serves as an embarrassment when discussing intimate topics for both patients and physicians. On the other hand, the vast majority of patients believe that it is appropriate for physicians to address sexual function within the context of a routine health assessment [38]. This confirms the need for publishing more research on SD in patients with different chronic illnesses, including MS.

82.5 % of our female patients have had a high probability of at least one sexual dysfunction. This prevalence is very high when compared to the most representative general population-base studies where: an estimated 43 % of women suffering from sexual problems according to the National Health and Social Life Survey (NHSLS) in the US [39] and 44.2 % of women did indicate at least one sexual problem as reported by Shifren and colleagues [40]. The prevalence of at least one SD in our patients is also higher than in previous studies of women with MS published by Barak and colleagues (50.5 %) [41] and Zorzon and colleagues (62.9 %) [20]. This could be due to different screening tools and a slightly different characteristic of study groups. In the research made by Barak and colleagues, the evaluation of SD was limited to patients with relapsing-remitting MS and in the study by Zorzon and colleagues, the mean EDSS score (2.6) was different than in our patients.

Three of the most common sexual problems (low desire, reduced sexual arousal and orgasm difficulties) were clearly higher in our patients than in the general population in the NHSLS: 57.7 versus 38.7 %, 47.3/48.4 %/45.2 %^1^ versus 26.1 % and 39.8 versus 20.5 %, respectively. The rates for decreased sexual desire, impaired arousal and difficulties in achieving orgasm in our group were within the upper range or slightly over the range estimated from reports by most of the other authors [13, 20–25]. Sexual pain was also not a frequent complaint in our patients (5.7 %) and this could explain why it was also rarely reported in any of the literature [19]. A higher prevalence of both decreased sexual desire (59.6 %) and orgasmic dysfunction (38.3 % with diminished orgasmic capacity and 12.8 % with a complete lack of an orgasm) were reported by Hulter and Lundberg [23] but they had examined women with more severe neurological impairments—a mean EDSS score of 6.5 compared to 5.2 in our patients. More recently, FSD in patients with MS, according to their hormonal status, were researched by Lombardi and colleagues [42]. Authors assessed 55 women using the Female Sexual Function Index, which is a well-designed and validated questionnaire for the evaluation of FSD [43]. Like in our study, they found desire to be the most affected domain of sexual functioning (57.4 %). However, more than one-third of all patients examined by Lombardi and colleagues had abnormal hormonal alterations; the correlations with sexual function were statistically insignificant, which further confirms the assumption of a multifactorial etiology of SD in patients with MS.

There are also different explanations for a higher prevalence of SD in our patients. First, they could be functionally more impaired than participants of other studies, as they were all recommended for rehabilitation. Second, we had used a validated tool for the assessment of SD, which can be more sensitive than author-designed questionnaires or interviews. Some other studies could also be biased as a result of using smaller study groups. And finally, we did not exclude patients with concomitant diseases like diabetes or cardiovascular factors that can independently cause SD.

Older patients scored lower in SFQ28 indicating more SD. That is consistent with a common finding for a higher prevalence of SD in older people [44, 45]. It is also surprising that over half of the patients claimed that the quality of their sexual life had not changed since the onset of MS. In another study, one by Hennessey and colleagues [21]—half of the female patients reported a deterioration of sexual activity while Zorzon and colleagues [20] reported that 48.6 % of women with MS had an unchanged quality of sexual life. This was further supported by much better results in most of the domains in SFQ28 compared to women who perceived that their sexual life had worsened. Both subgroups did not differ significantly in age and in duration of MS and EDSS score. It is understood that the course of MS is very unpredictable with different functional deterioration dynamics that are involved [46] so that the disease duration does not have to be a strong correlate with this (apart from the psychological influence of a longer-lasting status of being ill). In fact, the onset of the disease did not correlate significantly with their SFQ28 score. On the other hand, a woman’s perception of her sexual life was found to be dependent upon many different confounders like mood, quality of relationship or self-image, which makes this evaluation less reliable. However, even in patients perceiving that their sexual life did not change since the onset of MS, the prevalence of a highly probable sexual dysfunction was higher when compared to the general population of Poland [47] (44 % vs. 25 % for hypoactive sexual desire, 40 % vs. 12 % for decreased lubrication and 26 % vs. 10 % for anorgasmia, respectively).

It is commonly agreed that sexual response and relationship issues such as intimacy, attitudes and satisfaction strongly depend on each other [48]. According to McCabe and colleagues, 71 % of patients with MS and SD have marital problems [49]. It is worth emphasizing that in our study 81.3 % of females assessed their relationship with a partner from a positive standpoint. They scored much better in Desire, Arousal, Orgasm, Pain and Enjoyment domains of SFQ28 than patients who had a negative attitude towards their relationships. The comparison was insignificant only for the Partner Domain. This could seem surprising and suggested incoherence in their evaluation of the relationship between the interviewer and SFQ28 questionnaire. However, the SFQ28 Partner Domain contains two questions that relate to: (1) fear that the partner could search for another relation and, (2) perception of a partner’s negative feelings regarding their sexual activity. These questions do not evaluate the general quality of a relationship as it was obtained in an interview with patients. To conclude, the assessment of a patient’s relationship does not have to be coherent with SFQ28 Partner Domain.

The findings of our study confirm that SD is a serious problem in women suffering from MS. The frequency of SD, especially HSDD, FSAD, and difficulties in achieving orgasm and sexual enjoyment, is very high in patients with MS compared to the general population. A higher prevalence of SD in patients with MS could also be found with the use of more detailed and validated assessment methods. 97.8 % of our patients had never been asked before to discuss their sexuality in connection with their disease, which is similar to 94 % reported by Hulter and Lundberg [23] and 92.9 % reported by Zorzon et al. [20]. Medical professionals working with patients with MS should also pay more attention to their sexual concerns. This lack of interest in SD prevents patients with MS from receiving any professional therapy and could influence their quality of life negatively. These patients could also be diagnosed more accurately when using the appropriate screening tools in a routine examination of newly diagnosed MS during follow-up visits. Moreover, medical professionals working in the field of MS should be educated about the specificity of SD in patients with MS and how to screen them. The assessment of sexual function should also be implemented in all patients after the diagnosis of MS as SD may cause personal distress as well as relationship difficulties.

## Footnotes

1 The rates represent arousal dysfunction in three different dimensions: sensation/lubrication/cognitive, respectively.

## References

[1] Naeinian, M.R, Shaeiri, M.R., & Hosseini, F.S.:General Health and Quality of Life in Patients With Sexual Dysfunctions. Urol. J., **8,** 127–131 (2011). Retrieved from http://www.urologyjournal.org/index.php/uj/article/view/1023/560 21656471

[2] McCabe MP, Taleporos G (2003). Sexual esteem, sexual satisfaction, and sexual behavior among people with physical disability. Arch. Sex. Behav..

[3] Cyranowski JM, Bromberger J, Youk A, Matthews K, Kravitz HM, Powell LH (2004). Lifetime depression history and sexual function in women at midlife. Arch. Sex. Behav..

[4] Angst J (1998). Sexual problems in healthy and depressed persons. Int. Clin. Psychopharmacol..

[5] Schwarz ER, Rastogi S, Kapur V, Sulemanjee N, Rodriguez JJ (2006). Erectile dysfunction in heart failure patients. J. Am. Coll. Cardiol..

[6] Billups, K.L. Sexual dysfunction and cardiovascular disease: integrative concepts and strategies. Am. J. Cardiol, **96**(Suppl 12B), 57 M-61 M. (2005) doi: 10.1016/j.amjcard.2005.10.007 10.1016/j.amjcard.2005.10.007 16387569

[7] Schiel R, Muller UA (1999). Prevalence of sexual disorders in a selection-free diabetic population (JEVIN). Diabetes Res. Clin. Pract..

[8] Lundberg PO (1992). Sexual dysfunction in patients with neurological disorders. Annu Rev Sex Res.

[9] Lundberg PO, Brattberg A (1992). Sexual dysfunction in selected neurologic disorders: hypothalamo-pituitary disorders, epilepsy, myelopathies, polyneuropathies and sacral nerve lesions. Semin. Neurol..

[10] Lundberg PO (1981). Sexual dysfunctions in female patients with multiple sclerosis. Int. Rehabil. Med..

[11] Foley F, Sanders A (1997). Sexuality, multiple sclerosis and women. Mult. Scler. Manage..

[12] Foley F, Gimbel B (2005). Introduction to intimacy and sexuality in multiple sclerosis. MS. in. Focus..

[13] Minderhoud JM, Leemhuis JG, Kremer J, Laban E, Smits PM (1984). Sexual disturbances arising from multiple sclerosis. Acta. Neurol. Scand..

[14] Khan F, Pallant JF, Ng L, Whishaw M (2011). Sexual Dysfunction in Multiple Sclerosis. Sex. Disabil..

[15] Sipski M, Alexander CJ, Rosen RC (2001). Sexual arousal and orgasm in women: effects of spinal cord injury. Ann. Neurol..

[16] Gruenwald I, Vardi Y, Gartman I, Juven E, Sprecher E, Yarnitsky D, Miller A (2007). Sexual dysfunction in females with multiple sclerosis: quantitative sensory testing. Mult. Scler..

[17] Vardi Y, Gruenwald I, Sprecher E, Gertman I, Yarnitsky D (2000). Normative values for female genital sensation. Urology.

[18] Marrie R, Horwitz R, Cutter G, Tyry T, Campagnolo D, Vollmer T (2009). The burden of mental comorbidity in multiple sclerosis: frequent, underdiagnosed, and undertreated. Mult. Scler..

[19] Bronner G, Elran E, Golomb J, Korczyn AD (2010). Female sexuality in multiple sclerosis: the multidimensional nature of the problem and the intervention. Acta. Neurol. Scand..

[20] Zorzon M, Zivadinov R, Bosco A, MontiBagadin L, Moretti R, Bonfigli L, Cazzato G (1999). Sexual dysfunction in multiple sclerosis: a case-control study. I. Frequency and comparison of groups. Mult. Scler..

[21] Hennessey A, Robertson NP, Swingler R, Compston DAS (1999). Urinary, faecal and sexual dysfunction in patients with multiple sclerosis. J. Neurol..

[22] Valleroy ML, Kraft GH (1984). Sexual disturbances arising from multiple sclerosis. Arch. Phys. Med. Rehabil..

[23] Hulter BM, Lundberg PO (1995). Sexual function in women with advanced multiple sclerosis. J. Neurol. Neurosurg. Psychiatry.

[24] Demirkiran M, Sarica Y, Uguz S, Yerdelen D, Aslan K (2006). Multiple sclerosis patients with and without sexual dysfunction: are there any differences?. Mult. Scler..

[25] Dachille G, Ludovico GM, Pagliarulo G, Vestita G (2008). Sexual dysfunctions in multiple sclerosis. Minerva. Urol. Nefrol..

[26] World Health Organization (1994). The ICD-10 classification of mental and behavioural disorders: Diagnostic criteria for research.

[27] American Psychiatric Association (2000). Diagnostic and statistical manual of mental disorders (4th ed., text rev.).

[28] Fugl-Meyer KS, Lewis RW, Corona G, Hayes RD, Laumann EO, Moreira ED, Segraves T, Montorsi F, Basson R, Adaikan G, Becher E, Clayton A, Giuliano F, Sharlip I (2010). Definitions, classification, and epidemiology of sexual dysfunction. Sexual Medicine. Sexual Dysfunctions in Men and Women. 3rd International Consultation on Sexual Medicine.

[29] Hawkes CH, Giovannoni G (2010). The McDonald Criteria for Multiple Sclerosis: time for clarification. Mult Scler.

[30] Quirk, F.H., Heiman, J.R., Rosen, R.C., Laan, E., Smith, M.D., & Boolell, M.D. Development of the Sexual Function Questionnaire for Clinical Trials of Female Sexual Dysfunction. Journal of Women’s Health & Gender-based Medicine, **11**, 277–289 (2012). doi: 10.1089/152460902753668475 10.1089/152460902753668475 11988137

[31] Quirk FH, Haughie S, Symonds T (2005). The use of the Sexual Functioning Questionnaire as a screening tool for women with sexual dysfunction. J. Sex. Med..

[32] Symonds T, Abraham L, Bushmakin AG, Williams K, Martin M, Cappelleri JC (2012). Sexual Function Questionnaire: further Refinement and Validation. J. Sex. Med..

[33] Hatzichristou D, Rosen R, Derogatis L, Low WY, Meuleman E, Sadovsky R, Symonds T, Montorsi F, Basson R, Adaikan G, Becher E, Clayton A, Giuliano F, Sharlip I (2010). Clinical evaluation and symptom scales in men and women with sexual dysfunction. Sexual Medicine. Sexual Dysfunctions in Men and Women. 3rd International Consultation on Sexual Medicine.

[34] Slaski, S., Stefankiewicz, M. Psychometric validation of the Sexual Function Questionnaire in Poland. *Sex Disabil, 30*, 103–108. doi: 10.1007/s11195-011-9231-7 10.1007/s11195-011-9231-7 PMC3277819 22363092

[35] Kurtzke JF (1983). Rating neurologic impairment in multiple sclerosis: an expanded disability status scale (EDSS). Neurology.

[36] Schmidt EZ, Hofmann P, Niederwieser G, Kapfhammer HP, Bonelli RM (2005). Sexuality in multiple sclerosis. J. Neural. Transm..

[37] Nortvedt MW, Riise T, Myhr KM, Landtblom AM, Bakke A, Nyland HI (2001). Reduced quality of life among multiple sclerosis patients with sexual disturbance and bladder dysfunction. Mult. Scler..

[38] Vermillion S, Holmes M (1997). Sexual dysfunction in women. Prom. Car. Update Ob/Gyns.

[39] Laumann EO, Paik A, Rosen RC (1999). Sexual dysfunction in the United States: prevalence and predictors. JAMA.

[40] Shifren JL, Monz BU, Russo PA, Segreti A, Johannes CB (2008). Sexual problems and distress in United States women: prevalence and correlates. Obstet. Gynecol..

[41] Barak, Y., Achiron, A., Elizur, A., Gabbay, U., Noy, S., & Sarova-Pinhas, I. Sexual dysfunction in relapsing-remitting multiple sclerosis: magnetic resonance imaging, clinical, and psychological correlates. *J Psychiatry Neurosci,* **21**, 255–258.(1996) Retrieved from http://www.ncbi.nlm.nih.gov/pmc/articles/PMC1188782/?page=1 PMC1188782 8754594

[42] Lombardi G, Celso M, Bartelli M, Cilotti A, Del Popolo G (2011). Female sexual dysfunction and hormonal status in multiple sclerosis patients. J. Sex. Med..

[43] Meston, C.M. Validation of the Female Sexual Function Index (FSFI) in women with female orgasmic disorder and in women with hypoactive sexual desire disorder. *J Sex Marital Ther,* **29**, 39–46. (2003) Retrieved from http://www.ncbi.nlm.nih.gov/pmc/articles/PMC2872178/10.1080/713847100 PMC2872178 12519665

[44] Lindau ST, Schumm LP, Laumann EO, Levinson W, O’Muircheartaigh CA, Waite LJ (2007). A study of sexuality and health among older adults in the United States. N. Engl. J. Med..

[45] Laumann EO, Das A, Waite LJ (2008). Sexual dysfunction among older adults: prevalence and risk factors from a nationally-representative U.S. probability sample of men and women 57–85 years of age. J. Sex. Med..

[46] Lublin FD, Reingold SC (1996). Defining the clinical course of multiple sclerosis: results of an international survey. Neurology.

[47] Lew-Starowicz Z, Lew-Starowicz M (2002). Raport: seksualnosc Polakow 2002 [Report: sexuality of Poles 2002]. Przegl. Menop..

[48] Althof SE, Leiblum SR, Cevret-Measson M, Hartmann U, Levine SB, McCabe M, Wylie K (2005). Psychological and interpersonal dimensions of sexual function and dysfunction. J. Sex. Med..

[49] McCabe MP, McDonald E, Deeks AA, Vowels LM, Cobain MJ (1996). The impact of multiple sclerosis on sexuality and relationships. J. Sex. Res..

